# Basic and clinical immunology – 3027. Group of immune factors studied in relation to early development of allergic symptoms in an Indian subpopulation at one year of age

**DOI:** 10.1186/1939-4551-6-S1-P202

**Published:** 2013-04-23

**Authors:** Pooja Soneja, Meenu Singh

**Affiliations:** 1 Department of Pediatrics, Post Graduate Institute of Medical Education and Research, Chandigarh, India

## Background

It is speculated that Indian population is protected against allergies due to sufficient exposure to microbes and infections. Due to scarce information regarding birth cohort, principally, prompted us to follow a cohort for development of allergies and study the immune markers (cytokines and chemokine receptors) at one year of age.

## Methods

Healthy term newborns in families with h/o allergy were enrolled after informed consent at the time of birth. This Indian birth cohort of 99 babies with family history of allergy (75) and no history of allergy (24) was studied for minimum of 1.5 years for development of allergies with evaluation of chemokine receptors (CXCR3, CCR5, CCR3, CCR4, CCR8). Markers of Th1 (IFNγ, IL12p40) and Th2 (IL4, IL13) responses were also compared. Expression of molecules was studied using semi quantitative RT-PCR. The study was approved by the local Medical ethics review board.

## Results

Out of 75 infants in FH+, twenty (26.7%) developed allergic features. Positive maternal history was found as an independent risk factor. Expression levels were compared on the basis of family history (FH+ & FH-) and allergy development FH-ALG- (controls, babies with no FH and no development of allergic symptoms), FH+ALG+ (babies with positive FH and development of allergic symptoms), FH+ALG- (babies with positive FH and without development of allergic symptoms). On the basis of FH there was significant higher expression of CXCR3 and CCR5 in FH- while CCR8 expression was significantly higher in the FH+ group. IFN-γ was expressed at higher levels in FH- group whereas IL 13 was expressed at higher level in FH+ group. IL 13 was higher in the FH+ALG- and FH+ALG+ as compared to the FH-ALG- group but the difference was not significant. On applying multivariate analysis CXCR3, CCR5, CCR8 were found to be associated to allergy development.

## Conclusions

The findings support that allergy is an intrinsic phenomenon which differs with individual showing differential immune responses. Positive maternal history was found to be a strong risk factor for early allergy developments. Early infancy is an important period for development of allergies later in life and thus should be studied intensely.

